# Importance of a Laccase Gene (*Lcc1*) in the Development of *Ganoderma tsugae*

**DOI:** 10.3390/ijms19020471

**Published:** 2018-02-06

**Authors:** Wensong Jin, Jiahuan Li, Hongchang Feng, Si You, Liaoyuan Zhang, Justice Norvienyeku, Kaihui Hu, Shujing Sun, Zonghua Wang

**Affiliations:** 1 College of Life Sciences, Fujian Agriculture and Forestry University, Fuzhou 350002, China; jinws@fafu.edu.cn (W.J.); lijiahuan@fafu.edu.cn (J.L.); fenghcfafu@163.com (H.F.); yousifafu@163.com (S.Y.); zliaoyuan@mail.ecust.edu.cn (L.Z.); jk_norvienyeku@fafu.edu.cn (J.N.); hukh@fafu.edu.cn (K.H.); 2 Gutian Edible Fungi Research Institute, Fujian Agriculture and Forestry University, Gutian 352200, China; 3 Institute of Occean Science, Minjiang University, Fuzhou 350002, China

**Keywords:** *Ganoderma tsugae*, laccase, targeted gene replacement, over-expression, development

## Abstract

In this study, a novel laccase gene (*Lcc1*) from *Ganoderma tsugae* was isolated and its functions were characterized in detail. The results showed that *Lcc1* has the highest expression activity during mycelium development and fruit body maturation based on the analysis of *Lcc1* RNA transcripts at different developmental stages of *G. tsugae*. To investigate the exact contribution of *Lcc1* to mycelium and fruit body development in *G. tsugae*, *Lcc1* transgenic strains were constructed by targeted gene replacement and over-expression approaches. The results showed that the lignin degradation rate in *Lcc1* deletion mutant was much lower than the degradation efficiency of the wild-type (WT), over-expression and rescue strains. The lignin degradation activity of *G. tsugae* is dependent on *Lcc1* and the deletion of *Lcc1* exerted detrimental influences on the development of mycelium branch. Furthermore, the study uncovered that *Lcc1* deletion mutants generated much shorter pale grey fruit bodies, suggesting that *Lcc1* contributes directly to pigmentation and stipe elongation during fruit body development in *G. tsugae*. The information obtained in this study provides a novel and mechanistic insight into the specific role of *Lcc1* during growth and development of *G. tsugae*.

## 1. Introduction

Laccases (benzenediol: oxygen oxidoreductases, EC 1.10.3.2) are blue multi-copper oxidases that are involved in the oxidation of numerous aromatic substrates together with the reduction in molecular oxygen to water [1]. Laccases are distributed in a wide range of organisms [2], including plants, fungi, insects and bacteria and have been associated with diverse biological processes. Generally, laccases are classified into three categories according to the source, its physiological conditions and functions: (1) polymerization of monomers; (2) degradation of polymers; and (3) aromatic ring cleavage [3]. Multiple copies of laccase genes have been presented in white-rot fungi genome and are associated with crucial and diverse biological events, such as lignin degradation [4], pigmentation [5], mycelium development [6], fruit body formation [7], and detoxification of phenolic compounds [8], in the life cycle of the white-rot fungi. Fungal laccases have also been assigned other biological functions such as sporulation and plant pathogenesis [9]. Interestingly, in addition to laccase functioning in biological processes, isolated laccase protein itself exhibited some medicinal value, such as inhibitory activity towards human immunodeficiency virus (HIV)-1 reverse transcriptase [10,11,12,13]. However, due to unavailability of complete genome sequence data coupled with inefficient genetic manipulation methods, only a few of these genes have been functionally and experimentally characterized [14], especially those that function during mycelium and fruit body development.

*Ganoderma tsugae* as alternatively called Hemlock varnish shelf mushroom is a basidiomycete that belongs to the *Polyporaceae* of *Aphyllophorales*, representing one of the most important medicinal fungi that has been used as a folk remedy for the promotion of health and longevity in China, Japan, Korea, and other Asian countries [15]. Most of the previous studies relating to *G. tsugae* mainly focused on the isolation of its secondary metabolites and the characterization of their biological significance. Secondary metabolites extracted from *G. tsugae*, similar to those extracted from *Ganoderma lucidum*, have been shown to possess antioxidant activity, antitumor activity and anti-inflammatory properties [16]. Current information regarding the function of an individual or a specific gene (e.g., *laccase*) during growth and development of *G. tsugae* is very rare. Our previous work demonstrated that the levels of secreted laccase activity in the tested edible fungi closely correlated with the progression of their growth cycles [17]. Additional reports also showed that compared to other basidiomycete species, *G. tsugae* displayed higher levels of laccase activity in wheat bran [18]. A number of fungal laccase genes have been cloned and characterized in species belonging to the genus *Ganoderma* which includes *Ganoderma lucidum* [19], *Ganoderma* sp. En3 [20], and *Ganoderma fornicatum* [21]. Although these studies have proven that laccase genes from the genus *Ganoderma* can be forced expression in yeast, and their enzyme activities have been extensively investigated using in vitro assay systems. Knowledge on the in vivo functions of laccases in species of *Ganoderma*, especially *G. tsugae,* have not been explored, hence, questions regarding the specific roles played by laccases during growth and development of this important medicinal mushroom remain unanswered.

Targeted gene replacement has been variously applied as an efficient and fundamental approach for functional characterization of fungal genes over the years and entails several methods or techniques. Among these approaches, the use of the split-marker technique as targeted gene replacement strategy, which was initially applied in *Saccaromyces cerevisiae* [22], has gained popularity in filamentous fungi due to its conciseness, the implementation that only requires two rounds of PCR. More so, compared to the traditional homologous double recombination strategy, which has yields <1% to 20% possibility of deleting gene in filamentous fungi with maker-selected transformants, the split-marker strategy is highly efficient gene deletion technique, because antibiotic resistance gene can only be obtained through homologous recombination of overlapping selectable marker gene fragments in vivo [23]. Accumulating evidence confirmed the split-marker strategy as a potent tool for targeted deletion of genes in a wide variety of fungi species [23,24,25,26].

However, the split-marker method has not been used in the analysis of gene function of medicinal fungi. Thus, in this work, we aimed to address above issues by using the split-marker strategy to functionally characterize laccase gene in *G. tsugae* as well as to set up a system for functional characterization of medicinal fungal genes. This paper showed a successful functional analysis of laccase gene in *Ganoderma* strain using split-marker method for the first time. This research will provide great insights into the importance of laccase gene in genus *Ganoderma*. Our study also lays strong foundations for directional breeding of new species at the molecular level and the framework for studying the gene function of other medicinal mushrooms.

## 2. Results

### 2.1. Cloning and Analysis of the Lcc1 Sequence of G. tsugae

**Figure 1:**
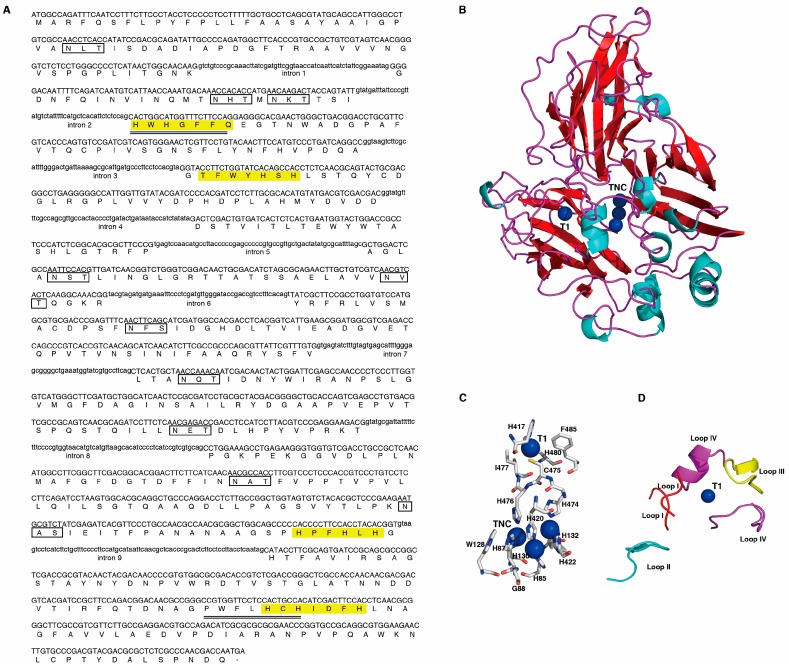
Nucleotide and deduced the amino acid sequence of *Lcc1* in *G. tsugae* and its structure model. (**A**) Nucleotide and deduced the amino acid sequence of *Lcc1* in *G. tsugae.* Double solid line, the amino acid sequence used to design degenerate primers; ten putative N-glycosylation sites are boxed; -, stop codon. The typical four ungapped conserved regions responsible for coordinating the four copper atoms at the active site of the Lcc1 are highlighted in yellow; (**B**) Proposed Lcc1 structure Model. The copper ions are depicted as blue spheres; (**C**) Residues contacting with one type-1 copper (T1), trinuclear cluster (TNC) that consists of one type-2 copper and two type-3 coppers or both (within 4 Å) in the Lcc1 model; (**D**) Four gapped loops that form a possible binding pocket for reducing substrates and T1 location.

A total of four ungapped conserved regions were identified in 64 fungal laccases by protein sequence alignment and were denoted as L1, L2, L3, and L4 for conserved region one, two, three and four, respectively [27]. The conserved regions L1 and L4 were selected and used for designing degenerate primers for amplifying putative laccase gene fragments in *G. tsugae* Gps1 strain (wild-type, WT). After amplification, 1611 bp DNA fragment was recovered and subjected to DNA sequencing. Subsequent multiple sequence alignment results showed that the DNA fragment obtained has high sequence identity (range from 80% to 99%) with laccase genes from other species of the genus *Ganoderma* (Figure S1A) and hence confirmed that the fragment is indeed part of a laccase gene in *G. tsugae*. Thermal asymmetric interlaced PCR (TAIL-PCR) was used to amplify the 5′- and 3′-flanking regions based on the DNA sequence of the fragment. The full-length of laccase gene (*Lcc1*) was successfully assembled from the genomic DNA. Further examinations showed that the assembled sequence comprises of 10 exons and 9 introns (Figure 1A). All the intron splice junctions correspond to the “GTNNG…C/TAG” rule of eukaryotic genomes. Additional sequence analysis results showed that the full-length *Lcc1* has an open reading frame (ORF) of 1566 bp which corresponds to 521 amino acids. Alignment of the deduced amino acid sequences of *Lcc1* with deduced amino acid sequences from other species of the genus *Ganoderma* showed high identity with laccase (ABK59825) from *Ganoderma tsugae* (99%), laccase (AAR82930) from *Ganoderma lucidum* (96%), laccase (ABK59827) from *Ganoderma fornicatum* (90%) and laccase (ADK55594) from *Ganoderma* sp. En3 (79%) (Figure S1B).

We further found that the Lcc1 protein sequence contains the typical four ungapped conserved regions, HWHGFFQ, TFWYHSH, HPFHLHG, and PWFLHCH, which are required to coordinate the four copper atoms at the active site of the Lcc1. To closely determine which residues within the four ungapped conserved regions directly mediate the interaction between the four copper ions and Lcc1, and to identify which possible polypeptides form binding pocket for substrates, based on the available laccase protein crystal structures from other basidiomycetes, we used Lcc1 full-length protein sequence as query sequence to search possible crystal structure templates in SWISS-MODEL template library (https://www.swissmodel.expasy.org/interactive), which possibly could be used as a template for Lcc1 to build a model by homolog-modelling strategy. Search results showed laccase from *Lentinus tigrinus* exhibits the highest identity with Lcc1 (74.10%). Therefore, a Lcc1 model was built on the basis of 2QT6 [28,29]. In the model, Lcc1 protein exhibit a molecular architecture organized in three sequentially arranged cupredoxin-like domains (Figure 1B). one type-1 copper (T1) is located in domain 3 and interacts with H417, C475, I477, H480, and F485 residues (Figure 1C). H417 is located within the ungapped conserved region L3, while C474, I477, and H480 all are located within the L4. The location of F485 is out of the four ungapped conserved regions and this residue may exert a major effect on the redox potential of the cupric ion [30]. Furthermore, a trinuclear cluster (TNC) consisting of one type-2 copper and two type-3 copper atoms is embedded between domains 1 and 3 in the Lcc1 model. The interactions between four ungapped conserved regions and TNC are mediated by H85, H87 and G88 residues within L1, by W128, H130, and H132 residues within L2, by H420 and H422 residues within L3, and by H474 and H476 residues within L4. In the catalytic process, T1 acquires electrons from reducing substrates and transfers electrons to TNC through an intramolecular electron transfer pathway. In the case of Lcc1, electron transfer pathway is composed of T1 ligand C475 and TNC ligands H474 as well as H476 (Figure 1C). In addition, in the Lcc1 model, four gapped loops were identified to form a possible binding pocket for reducing substrates (Figure 1D). Loop I is composed of two polypeptides and the sequences are RFPA and ACDPS, respectively. Loop II sequence is ANPSLGVMGF while loop III sequence is FGFDGTDFFI. The last loop also consists of two gapped polypeptides and the sequences are FPANANAAGSP and HIDFHLNAGF, respectively.

Current knowledge shows that most laccases of fungi origin are glycoproteins [31,32]. From our analysis, we identified ten putative N-glycosylation sites (Asn-X-Thr/Ser in which X is not Pro) in the protein sequence of the Lcc1 assembled from *G tsugae* in this study (Figure 1A).

### 2.2. Expression Profile of Lcc1 at Different Developmental Stages of G. tsugae

**Figure 2:**
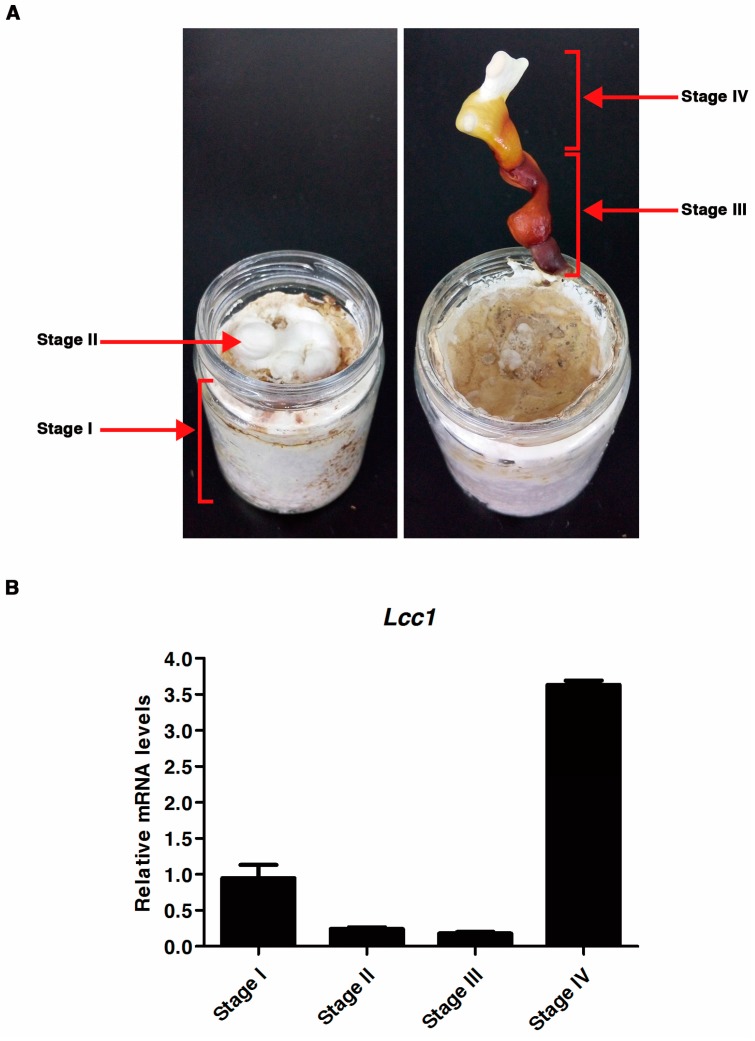
Analysis of *Lcc1* RNA expression levels during the life cycle of *G. tsugae*. (**A**) The developmental process of *G. tsugae* was divided into four stages: Stage I, mycelium; Stage II, primordium; Stage III, ripening stipe; Stage IV, fruit body maturation; (**B**) Total RNA was extracted from the Gps1 (WT) strain harvested at the following stages: Stage I (Day 14); Stage II (Day 16); Stage III (Day 40) and Stage IV (Day 40). The mRNA expression levels were analyzed by qRT-PCR and were normalized by using the relative mRNA ratio (*Lcc1*/*PRb2*). Values are means and bars indicate standard deviations (SDs) (*n* = 3).

To evaluate the expression activities of *Lcc1* at different developmental stages in the life cycle of *G. tsugae,* the wild-type (WT) Gps1 strain was cultured on a sawdust-substrate and tissues were collected from the cultured strain at four development stages (mycelium, primordium, ripening stipe and fruit body maturation). The expression activity of *Lcc1* in *G. tsugae* at defined stages of development (Figure 2A) was determined with quantitative real-time PCR (qRT-PCR) strategy using specific primers. Stage-specific *Lcc1* expression data generated from the qRT-PCR assay was analyzed by using Stage I *Lcc1* expression data as the reference condition for *G. tsugae* development and *RPb2* gene as the reference gene. Corresponding results obtained from this analysis showed that the expression of *Lcc1* was down-regulated by 3.9-fold at Stages II and 5.4-fold at Stage III. However, the expression of *Lcc1* was up-regulated by 3.8-fold at Stage IV (Figure 2B). More so, the expression of *Lcc1* was down-regulated at Stage II and Stage III, compared to the reference stage, suggesting that only a low amount of *Lcc1* is required for primordium formation and maintaining ripening stipe. Interestingly, *Lcc1* expression at Stage III was significantly down-regulated by 20.5-fold when compared to Stage IV, which suggests that during fruit body development, *Lcc1* plays a more important role in fruit body maturation than in maintaining ripening stipe. However, *Lcc1* expressed at Stage I implies that it may contribute to mycelium development and or lignin degradation, meanwhile the high expression activity displayed by *Lcc1* at Stage IV underscores its probable physiological importance in promoting fruit body maturation in *G. tsugae*.

### 2.3. Generation of G. tsugae Lcc1 Transgenic Strains

**Figure 3:**
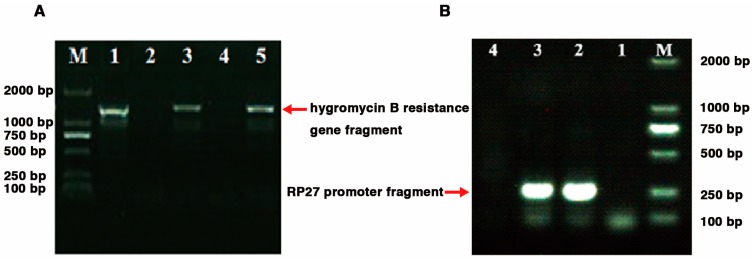
Identification of foreign DNA fragments in transformants selected by hygromycin B or zeocin. (**A**) Identification of foreign DNA fragments in transformants selected by hygromycin B. Amplification pattern obtained with primers (Ble-F and Ble-R) for the hygromycin B resistance gene fragment in the genomic DNA isolated from monokaryotic *G. tsugae* transformants and mated dikaryotic strain. Lane 1, A1 transformant of *Lcc1* knockout cassettes (Δ*Lcc1*-A1); lane 2, A1; lane 3, A9 transformant of *Lcc1* knockout cassettes (Δ*Lcc1*-A9); lane 4, A9; lane 5, dikaryotic strain Δ*Lcc1*-A19 which was generated by mating Δ*Lcc1*-A1 with Δ*Lcc1*-A9; (**B**) Identification of foreign DNA fragments in transformants selected by zeocin. Amplification pattern obtained with primers (RP27-F and RP27-R) for the RP27 promoter fragment in the genomic DNA isolated from Gps1 transformant and Δ*Lcc1*-A19 transformant*.* Lane 1, Gps1; lane 2, Gps1 transformant of pET11-*Lcc1* (*RP27*-*Lcc1*); lane 3, Δ*Lcc1*-A19 transformant of pET11-*Lcc1* (Δ*Lcc1*;*RP27*-*Lcc1*); lane 4, Δ*Lcc1*-A19.

In view of the differential expression pattern exhibited by *Lcc1* at different developmental stages of *G. tsugae*, we proceeded further to evaluate the exact roles played by *Lcc1* at Stage I and Stage IV, by generating *Lcc1* over-expression (*RP27*-*Lcc1*) strain, *Lcc1* knockout (Δ*Lcc1)* strain, and *Lcc1* complemented (Δ*Lcc1*;*RP27*-*Lcc1*) strain. Successful *Lcc1* deletion transformants harboring the hygromycin B resistance gene were selected on growth medium supplemented with hygromycin and confirmed with PCR. While over-expression and complementation transformants containing the zeocin resistance gene were selected on growth medium supplemented with zeocin and confirmed with PCR. We successfully identified two independent monokaryotic *Lcc1* knock-out mutants (Δ*Lcc1*-A1 and Δ*Lcc1*-A9) containing the hygromycin B resistance gene (Figure 3A). The transformation of an exogenous *Lcc1* fragment into both WT strain and dikaryotic Δ*Lcc1* mutant (Δ*Lcc1*-A19 was generated by mating Δ*Lcc1*-A1 with Δ*Lcc1*-A9) resulted in the generation of *Lcc1* over-expression (*RP27*-*Lcc1*) and complementation (Δ*Lcc1*;*RP27*-*Lcc1*) strains, respectively (Figure 3B).

### 2.4. Lcc1 Is Required for Mycelium Development

**Figure 4:**
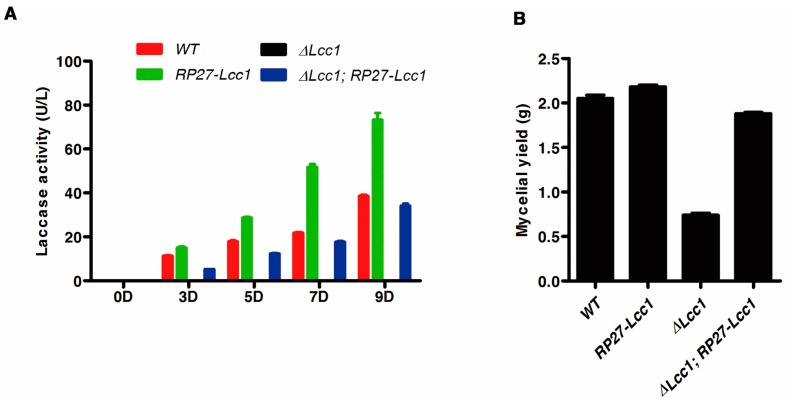

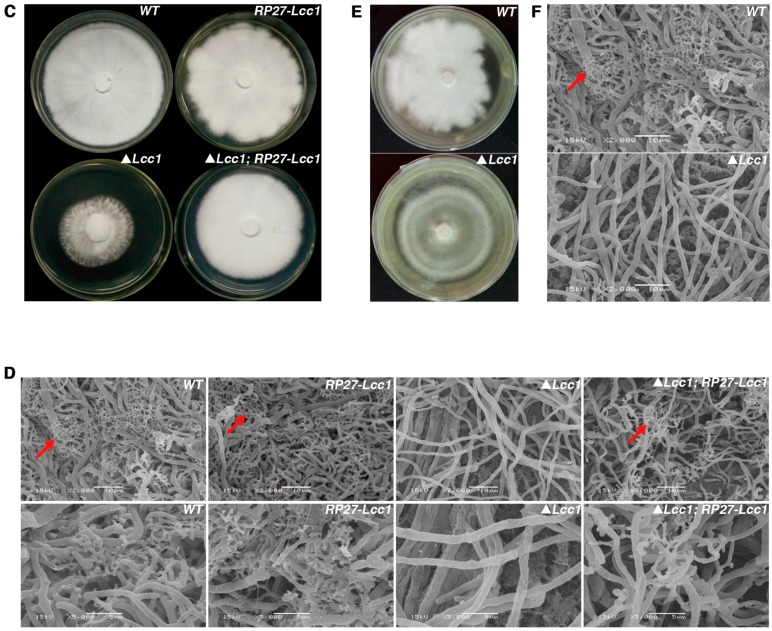
*Lcc1* is required for mycelium development. (**A**) Enzyme assays were used to determine the laccase activity of WT, *RP27*-*Lcc1*, Δ*Lcc1*, and Δ*Lcc1*;*RP27*-*Lcc1* strains cultured in liquid potato-dextrose broth (PDB) medium at the indicated time. Values are means and bars indicate SDs (*n* = 3). (**B**) WT, *RP27*-*Lcc1*, Δ*Lcc1*, and Δ*Lcc1*;*RP27*-*Lcc1* strains were cultured in 100 mL liquid PDB medium at 24 °C, 120 rpm for 10 days, and then the mycelia in the culture were recovered, dried and weighted. Values are means and bars indicate SDs (*n* = 3). (**C**) Colony morphology of WT, *RP27*-*Lcc1*, Δ*Lcc1*, and Δ*Lcc1*;*RP27*-*Lcc1* on potato–dextrose agar (PDA) plates; (**D**) Scanning Electron Microscope micrographs of samples from WT, *RP27-Lcc1*, Δ*Lcc1*, and Δ*Lcc1*;*RP27*-*Lcc1* strains cultured on PDA plates. The corresponding higher magnification micrographs were showed in the lower panels. Red arrows point to the mycelium branch; (**E**) Colony morphology of WT and Δ*Lcc1* on complete medium agar (CMA) plates; (**F**) Scanning Electron Microscope micrographs of samples from WT and Δ*Lcc1* strains cultured on CMA plates. The red arrow points to the mycelium branch.

Since *Lcc1* displayed a remarkable expression pattern during mycelium development stage, we speculated that *Lcc1* might be contributing to the progression of mycelium development and or lignin degradation. To test this hypothesis, we examined the existence of a possible linkage between secreted laccase activity and mycelium biomass generated in the WT, *RP27*-*Lcc1*, Δ*Lcc1*, and Δ*Lcc1*;*RP27*-*Lcc1* strains cultured in liquid potato-dextrose broth (PDB) medium. Results obtained from this examination showed that laccase activity increased progressively with culturing time in both WT and *RP27*-*Lcc1*, but there was no detectable activity of laccase in the Δ*Lcc1* strain at the investigated time of culturing (Figure 4A). Subsequent analysis conducted by weighing dried mycelium biomass collected from the respective strains at 10 days post-inoculation (dpi) further showed that the Δ*Lcc1* strain recorded the lowest mycelium biomass weight compared to the WT and *RP27*-*Lcc1* (Figure 4B). Additionally, we observed the re-introduction of *Lcc1* under RP27 promoter into the Δ*Lcc1* restored both laccase activity and mycelium biomass to the level of the WT (Figure 4A,B). These results suggested the laccase production in *G. tsugae* corresponds to mycelium biomass production.

Colony morphology of WT, *RP27*-*Lcc1,* Δ*Lcc1*, and Δ*Lcc1*;*RP27*-*Lcc1* strains were assayed on potato–dextrose agar (PDA) plates and, from this assay, we observed that the morphological characteristics exhibited by *RP27*-*Lcc1* and Δ*Lcc1*;*RP27*-*Lcc1* colonies were similar to the morphological features displayed by the WT colony, while the Δ*Lcc1* strain, on the other hand, produced relatively smaller colony than WT (Figure 4C and Figure S3A). To provide further insights into the factors accounting for the discrepancies observed in colony morphology of Δ*Lcc1* strain and the other three strains (WT, *RP27*-*Lcc1*, and Δ*Lcc1*;*RP27*-*Lcc1*) cultured on PDA plates, a scanning electronic microscope (SEM) was accordingly used to closely examine the morphology of mycelia produced by the respective strains. The outcome from these microscopy examinations showed that as shown in Figure 4D, colonies produced by the WT, *RP27*-*Lcc1*, and Δ*Lcc1*;*RP27*-*Lcc1* strains were composed of compact mycelium layer with numerous branches. Conversely, the colony of the Δ*Lcc1* strain consisted of sparse mycelium layer. More importantly, we also noticed that mycelia produced by Δ*Lcc1* were associated with pronounced branching defects and hence, produced almost non-branching mycelium compared to branching characteristics of WT, *RP27*-*Lcc1*, and Δ*Lcc1*;*RP27*-*Lcc1* strains. To ascertain whether a limited content of lignin in the PDA medium accounted for the morphological defect displayed by the Δ*Lcc1* colony, validation assay was performed by culturing the WT and Δ*Lcc1* strains on complete medium agar (CMA). Morphological and branching defects exhibited by the Δ*Lcc1* strain on CMA was similar to its growth on PDA medium (Figure 4F). However, there was no obvious difference in the growth rate of mycelium produced by the Δ*Lcc1* and the WT during growth on CMA plates (Figure 4E).

Taken together, our results suggested that *Lcc1* play multiple roles in *G. tsugae* through the degradation of lignin to facilitate the efficient liberation of nutrient to support normal mycelium growth as well as regulating important biological processes required for mycelium branch development.

### 2.5. Lcc1 Is Essential for G. tsugae Development

**Figure 5:**
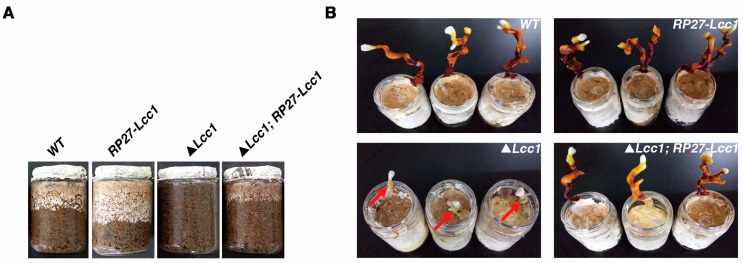

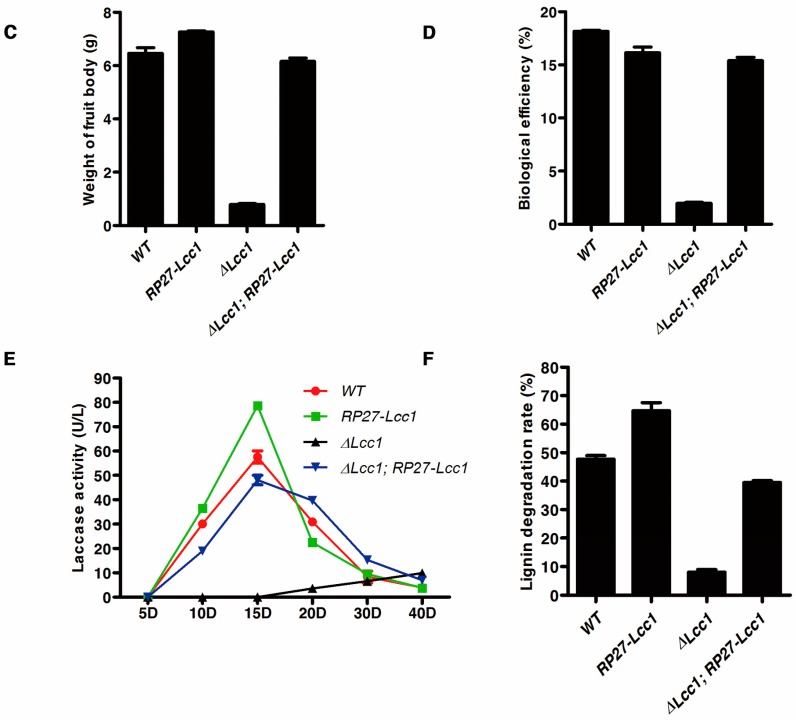
*Lcc1* is essential for *G. tsugae* development. (**A**) WT, *RP27*-*Lcc1*, Δ*Lcc1*, and Δ*Lcc1*;*RP27*-*Lcc1* strains were inoculated on sawdust-substrate and cultured at 24 °C. Pictures were taken at 11 days post-inoculation (dpi); (**B**) Pictures to show the morphology of fruit bodies of WT, *RP27*-*Lcc1*, Δ*Lcc1*, and Δ*Lcc1*;*RP27*-*Lcc1* at 40 dpi. Red arrows point to the stipe of Δ*Lcc1*; (**C**) WT, *RP27*-*Lcc1*, Δ*Lcc1*, and Δ*Lcc1*;*RP27*-*Lcc1* strains were inoculated on sawdust-substrate and cultured for total 40 days. Then, the fresh fruit bodies produced by them were harvested and weighted. Values are means and bars indicate SDs (*n* = 3); (**D**) After the fresh fruit bodies were weighted, the biological efficiency (%) was calculated by the equation: The biological efficiency (%) = the weight of fresh fruit bodies/the weight of cultivation sawdust-substrate × 100%. Values are means and bars indicate SDs (*n* = 3); (**E**) Enzyme assays were used to determine the activities of laccases in sawdust-substrate respectively produced by WT, *RP27*-*Lcc1*, Δ*Lcc1*, and Δ*Lcc1*;*RP27*-*Lcc1* at the indicated time. Values are means and bars indicate SDs (*n* = 3); (**F**) After the end of culturing, the lignin content in the sawdust-substrate was monitored by 72% sulfuric acid method and then the lignin degradation rate (%) was calculated. Values are means and bars indicate SDs (*n* = 3).

To investigate the influences of *Lcc1* on fruit body formation in *G. tsugae*, WT, *RP27*-*Lcc1*, Δ*Lcc1*, and Δ*Lcc1*;*RP27*-*Lcc1* strains were inoculated on sawdust-substrate. Growth initiation records generated from this study revealed that while, WT, *RP27*-*Lcc1*, and Δ*Lcc1*;*RP27*-*Lcc1* strains started to grow downwards at 6 dpi, the Δ*Lcc1* strain, on the other hand, started growing downwards at 18 dpi. Additional analysis also showed that the WT, *RP27*-*Lcc1*, and Δ*Lcc1*;*RP27*-*Lcc1* displayed similar mycelium growth rate characteristics (Figure 5A), in contrast to slow mycelium growth rate displayed by the Δ*Lcc1*. Although our investigations showed that the deletion of *Lcc1* in *G. tsugae* triggered a reduction in the size of fruit bodies produced by the Δ*Lcc1*, the size and pattern of fruit body formation in the *RP27-Lcc1* and Δ*Lcc1*;*RP27*-*Lcc1* strains were similar to fruit body formation characteristics observed in the WT strain (Figure 5B). We also evaluated and compared the color of fruit bodies produced by the WT, *RP27*-*Lcc1,* Δ*Lcc1*, and Δ*Lcc1*;*RP27*-*Lcc1* strains. Corresponding results derived from this color assessment assay showed that the WT, *RP27*-*Lcc1,* and Δ*Lcc1*;*RP27*-*Lcc1* all produced dark red fruit bodies while the Δ*Lcc1* produced pale grey ones (Figure 5B). The size and color of fruit bodies produced by the Δ*Lcc1* were not altered even after culturing for an extended period. At the end of culturing, the fresh fruit bodies were weighted, and then the biological efficiency was calculated. The average weight of fresh fruit bodies and the biological efficiency of the Δ*Lcc1* were much lower than values obtained from the WT, *RP27*-*Lcc1*, and Δ*Lcc1*;*RP27*-*Lcc1* strains (Figure 5C, D).

In addition, after analyzing data generated by examining laccase activity at different stages in the life cycle of *G. tsugae*, corresponding results showed that laccases activities recorded for WT, *RP27*-*Lcc1*, and Δ*Lcc1*;*RP27*-*Lcc1* at respective stage represented typical parabolic curves which reached peaks around at 15 dpi, while laccase activity of Δ*Lcc1* was only detectable at the later stage of culturing (Figure 5E). These data suggested that *Lcc1* directly contributes to the degradation of lignin at early stages of culturing. This conclusion was further confirmed by the observation that lignin degradation rate in the Δ*Lcc1* was much lower than the degradation efficiency of the WT, *RP27*-*Lcc1*, and Δ*Lcc1*;*RP27*-*Lcc1* strains (Figure 5F).

Taken together, our data strongly demonstrated that *Lcc1* is essential for lignin degradation during mycelium development and for pigments formation and stipe elongation during fruit body development of *G. tsugae*.

## 3. Discussion

Laccase, discovered by Yoshida in a lacquer tree for more than 130 years ago, has been well documented to be involved in degrading lignin in fungi [33]. Because of its high non-specific oxidation capacity, laccase has attracted intensive research attention regarding its utilization as a biocatalyst for a wide range of biotechnological applications, including pulp bleaching in the paper industry, dye decolorization, detoxification of environmental pollutants and wastewater treatment [34,35].

Generally, laccases of basidiomycetes secreted extracellularly from vegetative mycelia increase during fruiting and decrease when the fruit bodies mature. Laccases activities were found to be high in substrates used to cultivate basidiomycetes including *Pleurotus ostreatus*, *Agrocybe aegerita* and *Agaricus biporus* during vegetative growth phases [36,37,38], and dropped down in the process of fruit body formation. Therefore, it is thought that laccases produced by these basidiomycetes at vegetative growth stage degrade lignin and play a role in nutrient uptake to support the mycelium growth. With the progress of investigation on laccase in fungi, its other functions associated with pigment production [39] and fruiting body formation have been illuminated in detail. For example, the expression of *Lac4* gene isolated from *Volvariella volvacea* fruit body only can be detected at the later stage of colonization and reach the highest level at pinhead stage, but it maintained at certain higher expression level from the button to mature stage [32]. The pattern of *Lac4* transcription demonstrated that the laccase may play an important role in the process of fruit body development and melanin production of *V. volvacea*. Of course, there are some exceptions where *Lac4* gene can also activate the fruit body development in *Flammulina velutipes* and maintain the high expression level throughout the life cycle of *F. velutipes* [7]. In *A. auricular-judae*, *Lcc5* transcription level was found to be the highest among seven laccase genes analyzed and maintained at high expression level during fruit body formation and maturation, which suggested that *Lcc5* may play a major role in the process of fruit body development [40]. Fungal laccases have been assigned several physiological functions of relevance to mushroom cultivation. The high levels of laccase secreted by the mushroom strains had short produce cycle. Furthermore, laccases play a role in the fruit body morphogenesis of the mushroom and laccase gene is strongly expressed during the mushroom developmental cycle, which will contribute to improve the strain characteristics and shorten artificial cultivation period of mushroom, and lay a definite theoretical foundation for breeding high laccase-producing strains.

In the present work, *Lcc1* of *G. tsugae* was isolated and characterized. The deduced amino acid sequence of *Lcc1* has a high identity with protein sequence of laccases from genus *Ganoderma*, which suggests that *Lcc1* is indeed a laccase gene in *G. tsugae*. By analyzing its RNA expression levels during defined developmental stages in the life cycle of *G. tsugae*, *Lcc1* was found to be highly expressed during Stage I and Stage IV, suggesting that it might be involved in the regulation of physiological processes associated with these two stages. To ascertain the exact functions of *Lcc1* in *G. tsugae*, three kinds of transgenic strains including *RP27*-*Lcc1*, Δ*Lcc1*, and Δ*Lcc1*;*RP27*-*Lcc1* were generated by genetic manipulation approaches. The WT and *Lcc1* transgenic strains were analyzed to unravel the roles played by *Lcc1* during the development of *G. tsugae*. *RP27*-*Lcc1* and Δ*Lcc1*;*RP27*-*Lcc1* had characteristics (laccase activity, mycelium growth rate, mycelium biomass, colony morphology, and fruit body) similar to those of WT. But, compared to WT, Δ*Lcc1* exhibited much slower mycelium growth rate and produced much fewer amounts of laccase and mycelium biomass. The likely influence of laccase activity on mycelium growth rate was also reported in previous findings [6,41,42]. The production level of laccase was correlated positively with mycelium biomass of respective strains investigated in this study, and this observation is in agreement with previous findings that laccase production per milligram of mycelium was significantly lower for different monokaryotic and dikaryotic putative mutants producing low amounts of laccase than for the parent strain in *Pleurotus florida* [6]. In contrast to the findings that mutant producing low amounts of laccase lacked the ability to form fruit bodies due to their inability to efficiently colonize on the substrate [6], Δ*Lcc1* defective mutant generated in this study developed primordium and formed fruit bodies. Nonetheless, fruit bodies produced by the Δ*Lcc1* strain were smaller than those produced by the WT strain. It is worth stating that, irrespective of mycelium growth rate, the Δ*Lcc1* strain displayed pronounced defects in mycelium branch development when compared to WT; furthermore, the color of fruit bodies produced by the Δ*Lcc1* strain were characteristically pale grey while those produced by the WT were maroon; in addition, compared to the WT strain, the Δ*Lcc1* strain was associated with defective stipe elongation. The ligninase system, which has been confirmed to contribute to the mycelium development and fruit body maturation of *G. tsugae*, is often activated by the laccase expression in fungi. Although more than one laccase has been found during the life cycle of *G. tsugae* (Figure 5E), it seems that *Lcc1* is the predominant and functional laccase gene in *G. tsugae*. Therefore, the disruption of *Lcc1* gene resulted in the low expression of ligninase system and low production of mycelium and fruit body. In contrast to relatively extensive investigations on the induction of ligninase expression, the mechanism of ligninase activation by laccase remains largely unknown. In addition, the *Lcc1* gene is also crucial for *G. tsugae* to respond to various environmental conditions. 

**Figure 6:**
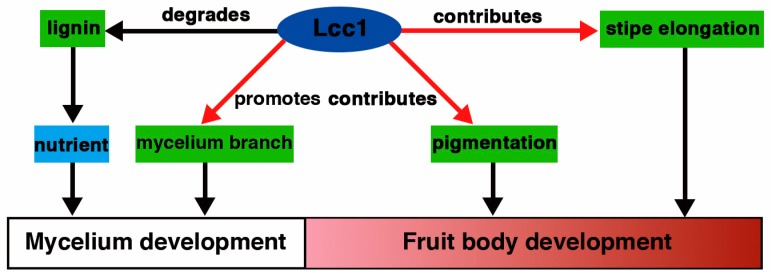
Proposed model to present the roles played by *Lcc1* during the development of *G. tsugae*. Laccases functioning in lignin degradation have been well documented and the *Lcc1* in *G. tsugae* was found to be involved in lignin degradation. In addition, the new roles, including promotion of mycelium branching, and contribution to pigmentation as well as stipe elongation, played by *Lcc1* were found in *G. tsugae* development (red arrows).

In reference to these results, we proposed an operational model for laccase activity in *G. tsugae* (Figure 6). In this model, we deduced that, during mycelium development, *Lcc1* degrades lignin into nutrient to support mycelium development and promotes the development of mycelium branch, and, during fruit body development, *Lcc1* directly contributes to pigment formation and to the stipe elongation.

## 4. Materials and Methods

### 4.1. Strains and Culture Conditions

The *G. tsugae* strain Gps1 (WT) used in this study was purchased from Agricultural Culture Collection of China (Beijing, China), and the strain number is ACCC51625. *RP27*-*Lcc1*, Δ*Lcc1*, and Δ*Lcc1*;*RP27*-*Lcc1* were generated from the Gps1 strain. Mycelia were cultured on potato–dextrose agar (PDA) plates or complete medium agar (CMA) plates at 24 °C, to monitor mycelium colony morphology and to prepare samples for scan electronic microscope analysis. The ingredients of PDA were potato 20%, glucose 2%, and agar 2%, natural pH. The ingredients of CMA were glucose 15.0 g, peptone 2.0 g, yeast extract 2.0 g, K_2_HPO_4_ 1.0 g, KH_2_PO_4_ 0.46 g, MgSO_4_ 0.5 g, agar 20.0 g and were dissolved in water up to 1 L. Mycelia used for laccase activity analysis and for mycelium biomass production analysis were cultured in 100 mL liquid potato-dextrose (PDB) medium (in exception of agar, the other ingredients were the same as PDA) at 24 °C, 120 rpm. For *G. tsugae* fruit body formation, the ingredients of sawdust-substrate (sawdust, wheat bran, corncob meal, sucrose, gypsum powder, calcium superphosphate) were mixed in the ratio of 78:17:2:1:1:1 and water was added to raise the final moisture content to 65%. The mixture was packed into the stopped jars (220 g/jar) and autoclaved at 121 °C for 3 h. Each jar was inoculated with one agar plug (diameter is 10 mm) that was inserted into the interior of the sawdust-substrate and then was incubated at 24 °C. Fruiting body formation conditions were created by opening the jars and transferring them to a culture chamber at 28 °C, with a relative humidity of 95%, and 50–100 lux light.

### 4.2. Molecular Biology Procedures and Plasmid Constructions

#### 4.2.1. Cloning the *Lcc1* Gene from *G. tsugae*

*G. tsugae* genomic DNA was isolated from the mycelia using the cetyltrimethyl ammonium bromide (CTAB) method [43]. The degenerate primer pairs (L1F and L4R) were used to amplify the DNA fragment of the putative laccase genes. All the primers used in this study were listed in Table S1. The DNA fragment was cloned into pMD-18T (Takara, Code No. D101A) and subjected to DNA sequencing. Sequence alignment was performed between the DNA fragment and previously found laccases from other species of the genus *Ganoderma*, including *Ganoderma tsugae* strain 1109, *Ganoderma lucidum*, *Ganoderma* sp. kk-02, *Ganoderma lucidum strain RZ*, *Ganoderma fornicatum*. Based on DNA sequencing results, the primers (GanoL1, GanoL2, GanoL3, and GanoL) and primers (GnoR1, GnoR2, GnoR3, and GanoR) were designed to amplify the 5′ and 3′ flanking sequence of *Lcc1* by TAIL-PCR with three rounds of reactions, respectively. Both 5′ and 3′ flanking sequence were cloned into pMD-18T and verified by DNA sequencing. The full length of *Lcc1* was assembled and analyzed.

#### 4.2.2. Analysis of *Lcc1* RNA Expression Levels during the *G. Tsugae* Life Cycle

To assess the RNA expression levels of *Lcc1* during the *G. tsugae* life cycle, four development stage samples (mycelium, primordium, ripening stipe, and fruit body maturation) were collected at 14 dpi for mycelium, at 16 dpi for primordium, at 40 dpi for ripening stipe and fruit body maturation, respectively, and their corresponding total RNA was extracted using Biozol RNA extraction kit (Biomiga, Catalog No. R1020-01), according to the manufacturer’s protocols. Two micrograms of total RNA were used to synthesize cDNA using a TransScript First-Strand cDNA Synthesis SuperMix (TransGen, Catalog No. AT301-03). qRT-PCR was conducted with primer pairs (Laccase-F and Laccase-R) using SYBR Premix Ex Taq^TM^ (Takara, Code No. RR420L). The relative *Lcc1* RNA expression levels were analyzed and normalized with *RPb2* RNA expression levels. Experiments were performed in triplicate and the results are presented as the mean values with SDs.

#### 4.2.3. Generation of *Lcc1* Knockout Cassettes

To generate *Lcc1* knockout cassettes, the 5′ UTR of *Lcc1* was amplified by self-formed adaptor PCR (SEFA-PCR) using primers (Sp1, Sp2, and Hemi-Sp3) with two rounds of PCR, while 3′ UTR of *Lcc1* was amplified by TAIL-PCR using primers (GanoR01, GanoR02, GanoR3, and S1010) with three rounds of PCR. 5′ UTR and 3′ UTR were cloned into pMD18-T (Takara, Code No. D101A) and verified by DNA sequencing respectively (5′ and 3′ UTR sequence provided in Tables S2 and S3, respectively). Based on the 5′ and 3′ UTR of *Lcc1* sequence and sequence of hygromycin B resistance gene, several new pairs of primers (Lac1-1F, Lac1-1R, Lac1-2F, Lac1-2R, YG-F, YG-R, HYG-F, and HYG-R) were designed to generate *Lcc1* knockout cassettes by the split-marker strategy with a couple of PCR. Finally, *Lcc1* knockout cassettes were purified and prepared for transformation.

#### 4.2.4. Generation of *Lcc1* Transgenic Strains by Genetic Manipulation Approaches

The strain *G. tsugae* was grown in PDA tube at 24 °C for 7 days and then was transferred to PDA plate for 5–7 days. The mycelial spawn from PDA plate was grown in a 250 mL flask containing 100 mL of PDB liquid medium at 24 °C on a rotary shaker at 110 rpm for 5 days. The mycelia were collected by centrifugation at 8000× *g* for 10 min. The collected mycelia were washed with sterile water and 0.6 mol/L mannitol solution and then blotted with sterile Whatman No.4 filter paper. Finally, with gentle agitation, the mycelia (approximately 0.2 g) were incubated for 4–5 h at 31 °C in 2 mL of 20 mg/mL lywallzyme (Shanghai Maokang Biological Technology Co, Ltd., China, Catalog No. MX7365-500MG) containing 0.6 M mannitol for preparing protoplasts. After incubation, these protoplasts were washed free of enzyme mannitol solution and transferred to regeneration medium (RM). One hundred microliters of protoplast suspension were poured onto regeneration medium agar (RMA) plates after re-suspension and dilution. The small hyphae were selected and transferred to new medium when the protoplasts regenerated. RM contained (per liter) 10 g glucose, 5 g maltose, 5 g yeast powder, 109.32 g mannitol. The ingredients of RMA were the same as that of RM with the addition of 20 g agar each plate. In this study, 87 colonies were picked by protoplast regeneration approach, in which 17 colonies were found to be monokaryotic strains. Six out of seventeen monokaryotic stains were randomly selected and subjected to incompatibility factor analysis. To determine the mating type among the monokaryotic strains, two small plugs (5 × 5 mm) of tested monokaryotic mycelia were placed closely together (1–2 cm apart) onto the PDA plate. The mated dikaryon strain was determined by the formation of clamp connections under a light microscope after incubation at 24 °C for 5–7 days. Our results showed that A1 mating type belongs to A1Bα1-β1; A5 and A7 mating type belong to A2Bα2-β2; A8 mating type belongs to A2Bα1-β2; and A9 and A15 mating type belong to A1Bα2-β2 (Figure S2A). A1, A5, A8 and A9 monokaryotic strains were selected for further colony growth rate analysis on PDA plates. A1 and A9 were finally selected due to their better growth characteristics (Figure S2B) and then *Lcc1* knockout cassettes were transformed into A1 and A9, respectively. Positive transformants were screened under the selection of hygromycin B (100 μg/mL) on PDA plates. By performing the mating between Δ*Lcc1*-A1 and Δ*Lcc1*-A9, Δ*Lcc1*-A19 strains were obtained under the selection of hygromycin B (200 μg/mL) on PDA plates. For generating *Lcc1* over-expression strain, cDNA sequence of *Lcc1* was sub-cloned into a pTE11 vector to create pTE11-*Lcc1* construct. Then the pTE11-*Lcc1* construct was transformed into protoplasts of Gps1 WT strain and finally, positive colonies were screened under the selection of zeocin (100 μg/mL) on PDA plates. For generating *Lcc1* complemented strains, the pTE11-*Lcc1* construct was transformed into protoplasts of Δ*Lcc1*-A19 strain and finally positive colonies were screened under the selection of zeocin (100 μg/mL) on PDA plates.

### 4.3. Enzyme Assays

The WT and *Lcc1* transgenic strains were cultured in liquid PDB medium. The laccase activity in the supernatant was determined with 2,2′-azinobis(3-ethylbenzothiazoline-6-sulfonic acid) (ABTS) as its substrate, according to the method by we previously described [17]. The activities of laccase in sawdust-substrate were monitored at the different stages during the life cycle of *G. tsugae*. After the treatment, the filtrate was recovered and centrifuged at 4 °C, at 5000× *g* for 30 min, and then the enzyme activity in the supernatant was determined with ABTS as its substrate.

### 4.4. Scan Electronic Microscope Analysis

Fresh mycelia were cut from cultivated strains, immediately followed by first fixing samples in 5% glutaraldehyde solution at 4 °C, for 4 h. Then fixed samples were rinsed with 0.2 M phosphate buffer for three times, each time 15 min. After the rinse, the samples were secondly fixed in 1% osmic acid for another 4 h at 4 °C. Fixed samples were washed by distilled water three times, each time 15 min, followed by dehydrated through a graded ethanol series, spending 15 min in each concentration: 50%, 70%, 80%, 90%, and absolute ethanol. The absolute ethanol rinse was repeated three times. The samples were then dried by the critical point drying method, mounted on SEM stubs using carbon tabs and coated with gold-palladium prior to imaging. Finally, samples were observed in an SEM and randomly selected fields were photographed.

### 4.5. Determining Lignin Degradation Rate by 72% Sulfuric Acid Method

The residual lignin in the sawdust-substrate was determined by 72% sulfuric acid method described previously by Geo. J. Ritter [44] with slight modifications. In brief, sawdust-substrate was collected and dried to constant weight at 60 °C. Then the substrate was ground and 1 g sample was weighed and transferred to a 100 mL Erlenmeyer flask. Fifteen milliliters of 72% sulfuric acid were added to the flask and mixed well with the sample. Then the flask was incubated at 18–20 °C for 2 h. After incubation, the mixed sample in the flask was transferred another 1000 mL flask coupling with the addition of 560 mL water followed by placing it into water-bath at 98 °C for 4 h. The hydrolyzed residue was filtered and washed free of acid by means of hot water, dried, and weighed. The lignin degradation rate was calculated by the equation below. Lignin degradation rate (%) = (1 − G_1_/G_2_) × 100%, where G_1_ was the weight of acid-insolvable and dried residue of the substrate which was used for cultivating WT strain or *Lcc1* transgenic strains; G_2_ was the weight of acid-insolvable and dried residue of the substrate which was not used for cultivating *G. tsugae*.

### 4.6. Nucleotide Sequence Accession Number

The coding DNA sequences of *G. tsugae Lcc1* has been deposited into the GenBank nucleotide sequence database with accession number KT166425.1 (https://www.ncbi.nlm.nih.gov/nuccore/KT166425.1). The *Lcc1* genomic DNA sequence, including its 5′ UTR and 3′ UTR sequences, has also been deposited into the GenBank nucleotide sequence database with accession number MG212669.

## 5. Conclusions

In summary, we successfully isolated and characterized one laccase gene (*Lcc1*) in *G. tsugae*, and further deployed targeted gene replacement and over-expression approaches as suitable genetic manipulation tools to generate *Lcc1* transgenic strains and proceeded further by analyzing the roles played by *Lcc1* during distinctive developmental stages in the life cycle of *G. tsugae*. We uncovered that *Lcc1* is not only involved in the degradation of lignin but also plays additional physiological roles that are associated with the development of *G. tsugae* and include the promotion of mycelium branching, pigment formation, and stipe elongation. Future efforts aimed at identifying natural endogenous substrates of Lcc1 and the related metabolic network will further help in uncovering the puzzle of *Lcc1* functions. *G. tsugae* has been used as an important medicinal fungi for a long history in Asian countries. Its secondary metabolites and polysaccharides have been regarded as the main medicinal components. However, it is intriguing to explore whether the Lcc1 protein in *G. tsugae* possesses such medicinal value as inhibitory activity towards HIV-1 reverse transcriptase and tumor cells.

## Supplementary Materials

The following are available online at http://www.mdpi.com/1422-0067/19/2/471/s1, Figure S1: Cloning and analysis of the *Lcc1* sequence of *G. tsugae*, Figure S2: Generating *Lcc1* transgenic strains, Figure S3: *Lcc1* is required for mycelium development, Table S1: Primers used in this study, Table S2: The 5’ UTR sequence of *Lcc1*, and Table S3: The 3’ UTR sequence of *Lcc1*.
