# Supplementary material for: Importance of a Laccase Gene (Lcc1) in the Development of Ganoderma tsugae
Source: Int J Mol Sci. 2018 Feb 6;19(2):471. doi: 10.3390/ijms19020471 (PMC5855693; doi:10.3390/ijms19020471)
Supplement: Supplementary file 1 [file ijms-19-00471-s001.docx]

**Supplementary information**

**Importance of a Laccase Gene (*Lcc1*) in the Development of *Ganoderma tsugae***

**Wensong Jin^1,2^, Jiahuan Li^1,2^, Hongchang Feng^1^, Si You^1^,** **Liaoyuan Zhang^1^, Justice Norvienyeku^1^, Kaihui Hu^1,2^, Shujing Sun^1,2*^, Zonghua Wang^1,3*^**

^1^College of Life Sciences, Fujian Agriculture and Forestry University, Fuzhou 350002, People’s Republic of China.

^2^Gutian Edible Fungi Research Institute, Fujian Agriculture and Forestry University, Gutian 352200, People's Republic of China.

^3^Institute of Occean Science, Minjiang University, Fuzhou 350002, People’s Republic of China.

* To whom correspondence should be addressed:

Shujing Sun, PhD

Tel: 86-591-83789492

Fax: 86-591-83789367

E-mail: shjsun2004@126.com

Zonghua Wang, PhD

Tel: 86-591-83761102

Fax: 86-591-83761102

E-mail: wangzh@fafu.edu.cn

**Supplementary Figures**

**
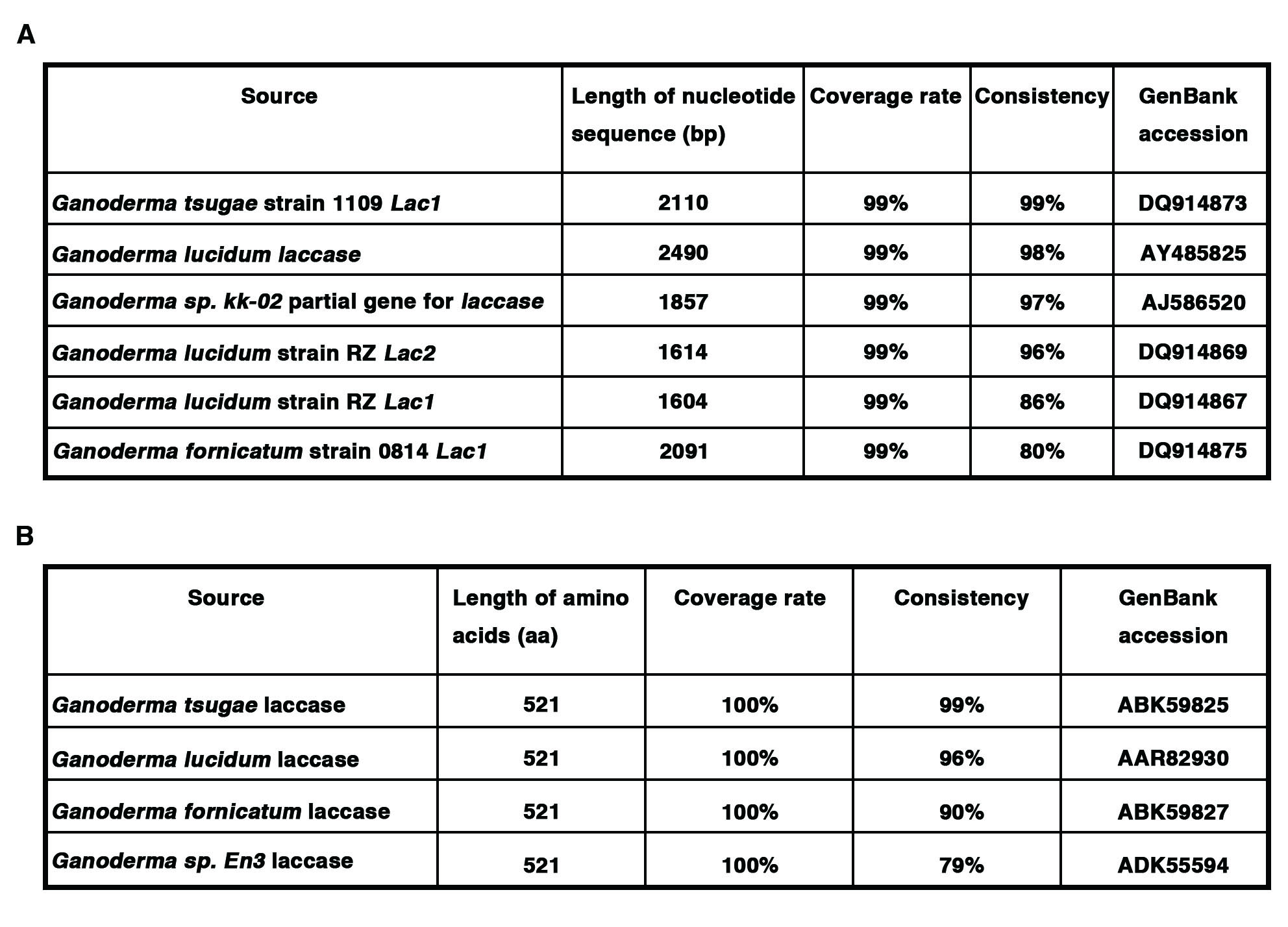
**

**Supplementary Figure S1** Cloning and analysis of the *Lcc1* sequence of *G. tsugae*.

(A) Nucleotide sequence alignment of the DNA fragment of *Lcc1* with previous found laccase genes from six species of genus *Ganderma*. Table to show the alignment results and related information.

(B) Amino acid sequence alignment of *Lcc1* with previous found laccase from four species of genus *Ganderma*. Table to show the alignment results and related information.


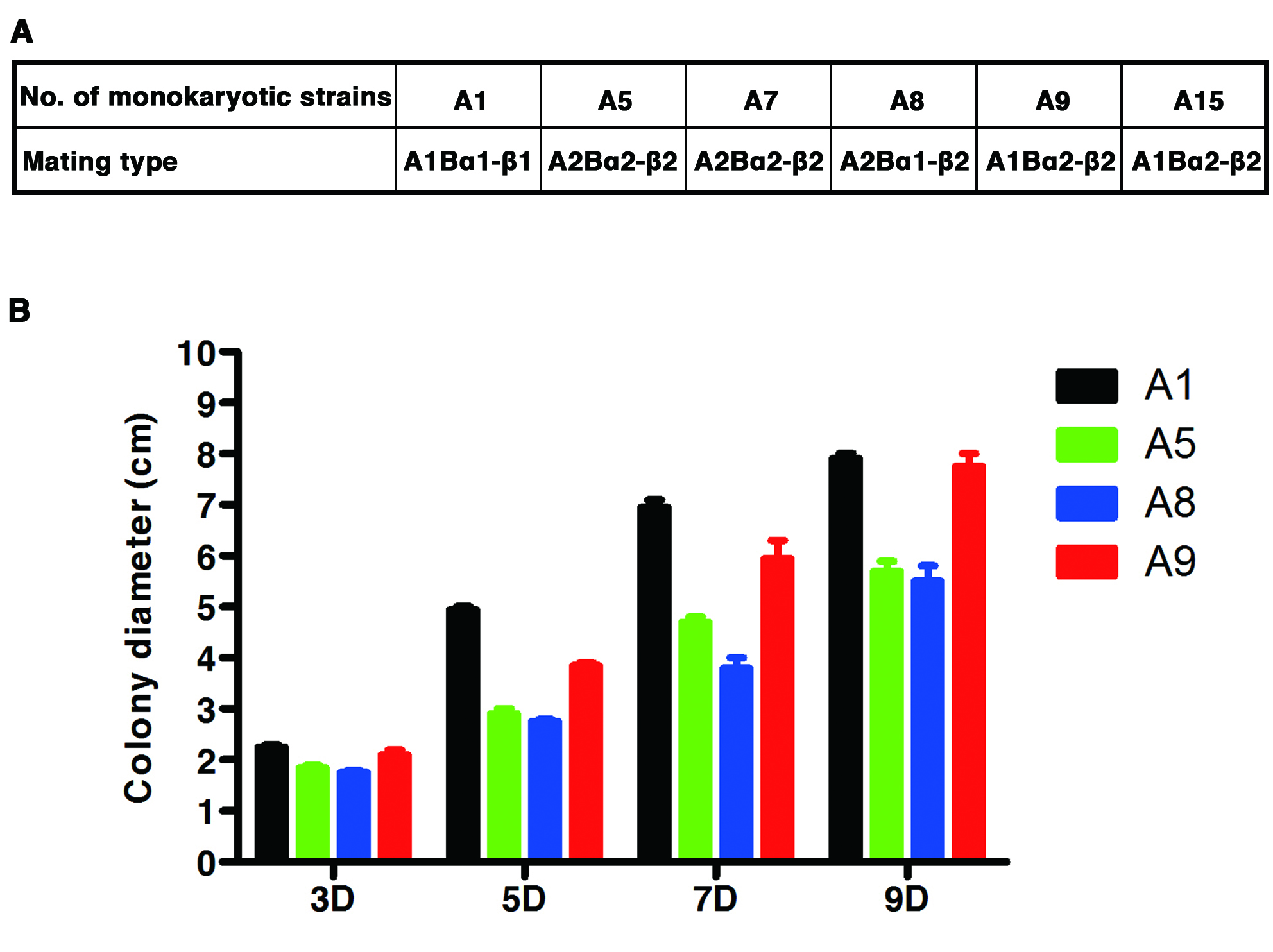


**Supplementary Figure S2** Generating *Lcc1* transgenic strains.

(A) Table to show the mating type of six monokaryotic strains.

(B) A1, A5, A8 and A9 monokaryotic strains were inoculated on PDA plates, and then the colonies diameter (cm) were measured at the indicated time. Values are means and bars indicate SDs (*n* = 3).


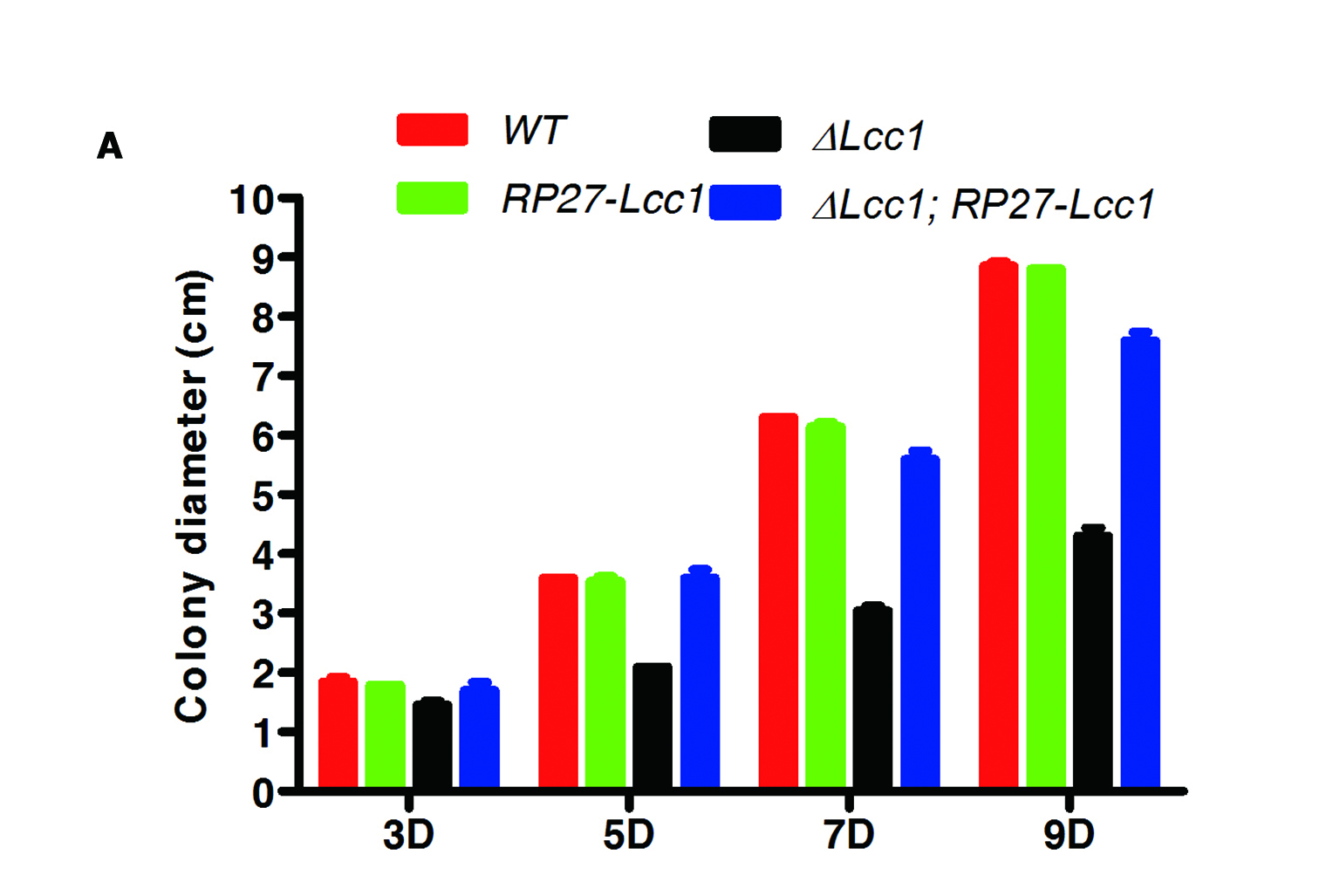


**Supplementary Figure S3** *Lcc1* is required for mycelium development.

(A) WT, *RP27-Lcc1,* Δ*Lcc1*, and Δ*Lcc1*; *RP27-Lcc1* strains were inoculated on PDA plates, and then the colonies diameter (cm) were measured at the indicated time. Values are means and bars indicate SDs (*n* = 3).

**Supplementary Tables**

**Supplementary Table S1** primers used in this study

| L1F | CA(C/T) TGGCA(C/T) GG(C/T) TTCTTCCA |
| --- | --- |
| L4R | TGGCA(A/G) TGGA(A/G) GAACCACGG |
| GanoL1 | GCGGAAGCGATAACTGTGAAAGG |
| GanoL2 | GAGGCGGTCCAGTACCATTCAGT |
| GanoL3 | CGTGCCCTCCTGGAAGAAACC |
| GanoL | ATGGC(G/C)AG(A/G)(T/C)T(C/T/G)CA |
| GnoR1 | ATGGCGTGCGACCCGAGTTT |
| GnoR2 | CAGCCCGTCACCGTCAACAG |
| GnoR3 | TTCAACGCTCACCCGCACTC |
| GanoR | CGCTCA(T/C)TG(A/G)TCGTT |
| Laccase-F | AAAGCCTGAGAAGGGTGGTGT |
| Laccase-R | GACGAAGGTGGCGTTGTTG |
| RPb2-F | GTGCCAGTATCATTCCGTTCC |
| RPb2-R | TTCGCCATCGTGTCCATC |
| Sp1 | TGGTATTATCAGTATCAGGGGTAGTGGC |
| Sp2 | TAGACATAACGGGAATAAATCATACAATAC |
| Hemi-Sp3 | GTTGCCAGTTATGAGGNNNNNNNNNGAGAC |
| GanoR01 | CAACGAGACCGACCTCCATCC |
| GanoR02 | TTCAACGCTCACCCGCACTC |
| GanoR3 | CGCCCAACGACCAATGAGC |
| S1010 | GGGATGACCA |
| Lac1-1F | TTCCAGATGACATTAGTGCCAG |
| Lac1-1R | TTGACCTCCACTAGCTCCAGCCAAGCCTTGGAGAGGACTGCTCGGTATC |
| Lac1-2F | GAATAGAGTAGATGCCGACCGCGGGTTCACAGGGACATTGATACTTATTTTGG |
| Lac1-2R | CGGGAGACAGGACAGGCTACT |
| YG-F | GATGTAGGAGGGCGTGGATATGTCCT |
| YG-R | GTATTGACCGATTCCTTGCGGTCCGAA |
| HYG-F | GGCTTGGCTGGAGCTAGTGGAGGTCAA |
| HYG-R | AACCCGCGGTCGGCATCTACTCTATTC |
| pTE11-*Lcc1*-F | CGGGATCCATGGCCAGATTTCAATCCTTTCT |
| pTE11-*Lcc1*-R | GACTAGTTCATTGGTCGTTGGGCGAGAGC |
| Ble-F | ATGGCCAAACTCACTTCTGCAGTC |
| Ble-R | GTCCTGTTCCTCAGCTACAAAATGG |
| RP27-F | CTCCAATACCCTGGTC |
| RP27-R | ATAGTCGGCTTTGTGC |

**Supplementary Table S2** the 5’ UTR sequence of *Lcc1*

| TCACTGGTCTTCCACGCCAACCAGCTATTCAATACCCTGTCCCGCTGACATGGGTGGCGACCTCAAGCAGTTGCTCTGAGGGCACTTGAGTTGTAACTTGCTCACATGGTGATGCAGGAAGGCGAGATAGCGAAAAATCCAGGACTCAGGGTTCCAAACCGCTCAGCCGCCGAACTTACTTAGCTATCATACGTGTCGTCTGTCAGCGATCGCCGCGACGATAAAATCGGAGGACAAGAAAAGCACGTATGGGCCCCTGAGTAGTCGGAGTCCTCGGGCCATCCATTTAGATTCTCGAATGCAACAACTCGAGTCATTGCTGCGGTTGTATGCAGAATGCGAAACATATGCCCGGCGCGCCGTACTGGTTGGGTGGCTCGGGAGTCCGGCTAGGAATAGGTCTACCTGTCATTTAGAGTGCGGCATGGAGGGATTCTAAATTGGGCGAGGACTCACGGATTGGCGACGATACTATGACGCAAGGTTCAAAACTAGAGATGCTGGACGAGAGCAATGGGTGAGAACTCCCGTCGTGGGTAGGTGGGCGGAACCTTTGACAGATTCGTCGCCCAAGTGTCTCTAGGACGGTGCTGCGTTTCAACCTGATTTTGGCCTACATACATGGGACTGAACATGACATATTTCCAGATGACATTAGTGCCAGTGGAGGTGGCTCACTGGATGGCCATACGGGGGAGGTGGTGGCGGAGAGCATGCGCAAGGGTGGCTATCTACAAGCGCCCGCATGCGTTGCCGTCGAAACAAGCGCCTGTGGCAGAGCATGTCCGTTTTTGGCAGCCACTGCGTCTTTTGTTCGCAGCGTTGACTCGGACTTGGACTAGCCCCCTCACCTTGACTCCCCTGCATGTTGTGGTCGCAGACATGACTAGCACAGCTCAGCCTTTTAATGAACACTGCGCTCAGTGTTAGCAGGATCCATCATAGGTTTCGTCAGCGTTGGCCGCAGCCACCGTCCGAATGCACTTCGGATGCCGCGGAACCATTGGTGCGCCCATTCTCCATTGCGACCTTCGCACACATAGCAGCTTAGCAAGCTAGTTCTTTATGACCGGCATGCACTCTTACGAACTGCGCACAACCGATATGTATAGCTCACACTGTCGCCTGTCCAGGTCCGAGACAAATGGATTCAGGTACACTCCTACCGCCAGTGGCGGTCCCGAAATGCGTCAAGTCGGAAATCAAACCCTACAACGCTCCACAGGTCATGAAACCAAAGTTACACTGGAGGTAAGGTTCTGTCCTGTATTGTAGGGAAGCTCTCACATATGTCGTAGAGGTCGCCCGACAACCGCGTGCTATGCCACAGTGGTATGCGCCACCAAACTTGGACAGCGTCCCGTATCCATACAAGCCACCGTATTAGCATAGCTATGGCGGGCCGAGACGGTTGCGCTGACGCGATCGCTTTTTCCGAAGAGTATAAAAGACATGGGTATGCGGGGGAAGCCATCCCAACCCAACTCCTCACTCGATACCGAGCAGTCCTCTCCAAGTGCGACTGCC |
| --- |

**Supplementary Table S3** the 3’ UTR sequence of *Lcc1*

| GCGCTGGATGTTGAATCGGTGCGCGCGACTTCGCGGGCCGAAGTTTGCACAGGGACATTGATACTTATTTTGGATTATACCTTACGTTCGTTCTTTAGTCCGAGTGCGGGTGAGCGTTGAATGCCTGAACAGTCCTACTCTTGTAATTACCATATTCTTATTTGTAGTTCGTAGTCTAAGTCAATACCAGTCGATGGTTATCGCTTCTTCTATCAGCTGGGCGACGTCTACGCAGACAAGAAGTCCGCTTTCCTCCAAGCCTCTAGCCAAGTCCGCGCCCGCTCTTTGTCCAGCTGCGGTAGCCGTGCCAACGACTCGGATACCCGCACCGCCTTATCCGTTGCGATGAACACGAGGCCCATCGCCTCCCTATCCGGCGCGCCCTCGGTCGTCTCGCCCTGCGCCTTGTAGAATGACAGAAGCCGAAGCGCGGGGTTAAGGCGCATCGCCTCGACTTCTTCCGCGGAGCCTCGCTCGACACCGCCTTCAGGCGCGGAGAGCCACTCGGCGTACGGCACGAGAGGGATATCGAGTTCCTGTGCGAGTGGAGCGAGCAGTGTGGTCCACGATACGAGATGTACGATCGGCTCAGACGAGCGGCGCATCTCCACGAACGCCCGCGCGGCCTCGTACGCCGGGACCCATGCCACTCTCTAAGAATACAGAAATTGATTGAGTCCGATAGCCTTGGGGGGAACCGTAAAGCAGATACTGCTTACCCCATTGTGCTTGGGGAGGCATTTCTGGAAGAGCGCGGATTTGACGAGGGACGGGAACCACTCGCGCTCGTTCCAGTAGCCTGTCCTGTCTCCCGCGACCTGTCCGAGGCGCATCACGACGGCATGCACGCCACGCCGCTGGGTAACGTTTTGCAGAATGCGCTCCGCGACCCACTTGCTCTCCGAATACCCGGATCCGAAAGGCGACGCCGGGTCGTCGATCGGTTTTTCGGGCACCGGCGCGGCGATCTTGCAGTCTAGTAAGCGGATGGAGGTCGGTCTCGTTGGGATGGAGTGCGGGTGAGCGTTGAA |
| --- |
